# Genetic Risk Factors Related to Coronary Artery Disease and Role of Transforming Growth Factor Beta 1 Polymorphisms

**DOI:** 10.3390/genes14071425

**Published:** 2023-07-10

**Authors:** Damian Malinowski, Oliwia Bochniak, Katarzyna Luterek-Puszyńska, Michał Puszyński, Andrzej Pawlik

**Affiliations:** 1 Department of Pharmacokinetics and Therapeutic Drug Monitoring, Pomeranian Medical University, 70-111 Szczecin, Poland; damian.malinowski@pum.edu.pl; 2 Department of Physiology, Pomeranian Medical University, 70-111 Szczecin, Poland; oliwia.bochniak@pum.edu.pl; 3 Department of Urology and Oncological Urology, Regional Specialist Hospital in Szczecin, 71-455 Szczecin, Poland; katarzynaluterek@gmail.com (K.L.-P.); michalpuszynski@gmail.com (M.P.)

**Keywords:** unstable angina, coronary artery disease, CAD, genetics, SNP, GWAS, TGFβ, TGFBR

## Abstract

Coronary artery disease (CAD) is one of the leading causes of mortality globally and has long been known to be heritable; however, the specific genetic factors involved have yet to be identified. Recent advances have started to unravel the genetic architecture of this disease and set high expectations about the future use of novel susceptibility variants for its prevention, diagnosis, and treatment. In the past decade, there has been major progress in this area. New tools, like common variant association studies, genome-wide association studies, meta-analyses, and genetic risk scores, allow a better understanding of the genetic risk factors driving CAD. In recent years, researchers have conducted further studies that confirmed the role of numerous genetic factors in the development of CAD. These include genes that affect lipid and carbohydrate metabolism, regulate the function of the endothelium and vascular smooth muscles, influence the coagulation system, or affect the immune system. Many CAD-associated single-nucleotide polymorphisms have been identified, although many of their functions are largely unknown. The inflammatory process that occurs in the coronary vessels is very important in the development of CAD. One important mediator of inflammation is TGFβ1. TGFβ1 plays an important role in the processes leading to CAD, such as by stimulating macrophage and fibroblast chemotaxis, as well as increasing extracellular matrix synthesis. This review discusses the genetic risk factors related to the development of CAD, with a particular focus on polymorphisms of the transforming growth factor β (TGFβ) gene and its receptor.

## 1. Introduction

Atherosclerosis is associated with several risk factors that are mostly preventable and manageable, such as hypertension, hypercholesterolemia, smoking, and diabetes. Several unavoidable risk factors, such as advanced age, gender, and family history of early myocardial infarction, also have a significant impact. The complex process of atherosclerosis begins early in life. It is thought to initiate with dysfunction of the endothelial cells that line the coronary arteries; these cells are no longer able to appropriately regulate vascular tone, leading to a narrowing of the vessel and obstruction of the blood flow [1,2].

Studies in recent years have shown that atherosclerosis is a chronic inflammatory process that takes place in the vessels, causing endothelial damage and dysfunction [1].

The ongoing inflammatory process causes accumulation of atherogenic lipoproteins in the vessel wall, leading to the development of atherosclerotic plaque, the instability of which increases the risk of its rupture, which can lead to acute coronary syndromes [2].

Numerous cells of the immune system, such as leukocytes, macrophages, and lymphocytes, are involved in this process, and produce mediators that increase and maintain inflammation. These include a great number of cytokines, chemokines, metalloproteinases, and growth factors.

The role of inflammation in the development of atherosclerosis was confirmed based on the effectiveness of statins in reducing the risk of developing coronary artery disease. The results of studies suggest that most of the beneficial effects of statins are due to the reduction in inflammation in the vasculature, not just their lipid-lowering effects [3].

A major role in the development of the atherosclerotic process is played by endothelium dysfunction. Ongoing inflammation within the endothelium causes loss of its normal functions, resulting in excessive platelet aggregation and the formation of local thrombotic lesions [3]. In addition, there is a decrease in the secretion of mediators with vasodilatory properties, such as nitric oxide, and an increase in the synthesis of vasoconstrictive mediators, such as endothelin. In addition, there is a subendothelial accumulation of lipoproteins, which undergo oxidation and aggregation into large complexes that initiate the formation of atherosclerotic plaque [3,4].

Oxidized lipoproteins are absorbed by macrophages located in the endothelium, which secrete numerous cytokines and adhesion factors, such as intercellular adhesion molecule 1 (ICAM-1), E-selectin, and vascular cell adhesion molecule 1 (VCAM-1) [3,4]. This process additionally increases inflammation in the vessel wall and the development of atherosclerotic lesions. Due to the presence of adhesion molecules, circulating monocytes can adhere to the endothelium and differentiate into macrophages, which secrete a number of pro-inflammatory cytokines involved in both the development of inflammation and the atherosclerotic process (IL-1α, IL-1β, IL-6, IL-15, IL-18, TNF-α), as well as anti-inflammatory cytokines (like interleukin 4, 10, and 13, and transforming growth factor β (TGF-β)), which can inhibit both the development of inflammation within the vessel endothelium and the atherosclerotic process [3,4].

The development of inflammation and atherosclerosis is aggravated by an imbalance between the production of pro- and anti-inflammatory mediators. An important role in atherosclerotic plaque formation is played by macrophages, which absorb oxidized low-density lipoprotein C (LDL-C) to form cholesterol-containing foam cells. Under the influence of inflammation, they undergo apoptosis, which stimulates the migration of vascular smooth muscle cells into the intima [2,3,4]. Ongoing inflammation makes the atherosclerotic plaque unstable and prone to rupture, increasing the risk of thrombosis. Also, hypoxia causes the development of neovascularization, increasing the instability of atherosclerotic plaques. The inflammatory process also enhances the secretion of metalloproteinases, which cause collagen degradation, making the atherosclerotic plaque unstable.

Previous studies have demonstrated the influence of many factors on the development of the atherosclerotic process [2,3]. Among the main risk factors for atherosclerosis are disorders related to lipid metabolism, carbohydrate metabolism, and excessive activity in the sympathetic nervous system. Normally, in healthy subjects, the activity of the parasympathetic and sympathetic nervous systems are in equilibrium. The main mediator of the sympathetic nervous system—acetylcholine—inhibits the activity of macrophages, blocking their secretion of pro-inflammatory cytokines. Stimulation of the sympathetic nervous system blocks parasympathetic influences, resulting in excessive secretion of vasoconstrictive mediators, such as endothelin, and decreased secretion of vasodilatory mediators, such as nitric oxide.

Coronary artery disease (CAD), which is commonly referred to as coronary heart disease (CHD) or ischemic heart disease (IHD), is triggered by the formation of plaques in coronary arteries, which play a crucial role in the supply of oxygenated blood to the heart muscle [3]. Increased circulating LDL cholesterol, increased triglyceride-rich lipoproteins, and reduced levels of high-density lipoprotein (HDL) cholesterol in the blood are associated with CAD risk. These risk factors can be merged to identify subsets of the population that are at an increased risk of CAD. Moreover, CAD is one of the main causes of death worldwide, regardless of nationality or ethnic group. Increasingly, recent studies have demonstrated its complexity and tried to understand the genetic factors underlying it.

## 2. CAD Genetic Background

An individual’s risk of developing CAD is modulated by a set of genetic and lifestyle factors, as with many other complex diseases. Clinical observations from the mid-20th century have supported the belief that CAD risk is heritable [4]. In the Framingham Heart Study, a family history of cardiovascular disease in a parent or sibling was a strong predictor of CAD [5]. These studies laid the groundwork for applying new tools of human genetics to understand the genetic basis of CAD and translating these findings into clinical practice. The familial risk of CAD was first described in studies involving twins and prospective cohorts [6,7]. The Swedish Twins Registry involved nearly 21,000 individuals who were observed for more than 35 years; the inheritance of fatal CAD events among this group was calculated to be 0.57 and 0.38 for males and females, respectively. It is worth noting that hereditary effects are most pronounced in younger individuals [8]. 

Case–control studies examining the likelihood of CAD compared to healthy controls have revealed many risk factors in recent years. However, this scientific approach is not always suitable for detecting new genetic risk factors. The chromosome 9p21 locus is the most widely replicated genetic CAD risk locus identified to date, with an estimated 15% to 35% increased risk in carriers of the variant allele in prospective population-based studies and case–control studies [9]. The exact mechanism underlying the 9p21 association remains unknown. Preliminary evidence suggests that 9p21 variants alter the expression of antisense non-coding RNA at the INK4 (inhibitors of cyclin-dependent kinase 4) locus (ANRIL), thereby altering the activity of two nearby cyclin-dependent kinase inhibitors (CDKN2A and CDKN2B) involved in cell cycle regulation and cellular proliferation [10]. ANRIL is a well-defined genetic risk locus in Chr9p21 that is associated with endothelial dysfunction and triggering atherosclerotic processes [11]. A recent cohort study showed that variation on chromosome 9p21 has no significant association with CAD risk [12].

## 3. Role of Single-Nucleotide Polymorphisms in CAD

Cardiovascular monogenic diseases such as vascular disorders, inherited cardiomyopathies, and rhythm disorders are mostly associated with large, rare DNA variants. In contrast, other complex diseases may be influenced by common DNA variants at numerous, scattered loci distributed throughout the entire genome. Single-nucleotide variations typically have small effects and do not drive classic Mendelian inheritance patterns (familial inheritance). They also require large-scale, population-based, association studies for discovery. 

A single-nucleotide polymorphism (SNP) is a common genetic variation at a single position in a DNA sequence among individuals (occurs at a specific position in the genome), being present in more than 1% of the population [13]. A SNP may be a marker of disease susceptibility. 

The sequencing of the human genome has played a key role in understanding the genes associated with CAD. Advances in understanding the genetics of CAD have been stimulated by the development of DNA microarray technology using chips containing up to one million DNA markers consisting of SNPs. These have provided the basis for association studies, known as genome-wide association studies (GWAS) [14]. Genetic association studies examine the correlation between disease and chromosomal abnormalities and gene mutations, such as the aforementioned SNPs, but also variable number tandem repeats (VNTRs) and copy number variations (CNVs) to identify risk or protective alleles that play a role in the development of a specific disease. An abnormal frequency of a risk allele or genotype in affected individuals with a specific disease suggests that the analysed variant increases the risk of a specific disease [15]. GWAS have been used to create genetic risk scores to improve CAD risk prediction [2]. These studies use high-throughput genotyping technologies to map thousands of SNPs in the human genome and correlate them with clinical conditions or measurable traits. GWAS are very useful in discovering genetic variants related to different diseases (like CAD); however, they have some limitations. 

These studies are based on genes with a known or suspected role in defining the phenotype in question and do not provide new information on biological pathways linked to the disease. Candidate gene associations generally cannot be replicated for several reasons, one of these may be a too low statistical power related to inadequate study group size [14]. The first association of common variants (CVAS) for ischemic heart disease was published by three independent teams in 2007, and they described common variants at the 9p21 locus associated with a ~30% increase in CAD risk [16,17,18]. Subsequent studies have both replicated this finding and extended the link to other vascular phenotypes, including carotid atherosclerosis [19], peripheral artery disease [20], and stroke [21].

SNP genotyping will always be associated with false positives and false negatives, differences in the selected study populations, and simple genotyping errors [22]. Meta-analyses partially address some of these limitations. They combine the results of single-gene association studies, providing an excellent opportunity to observe the role of a single genetic variation in a more heterogeneous and larger population. Over the past 10–15 years, meta-analyses have looked at a wide range of conditions associated with heart disease, including CAD itself [23,24,25,26]. Unfortunately, meta-analyses, like GWAS, can be biased by population stratification, variability in control group selection, different study approaches, or different scales of biochemical values. Most SNPs identified by GWAS still have very insignificant effects on disease outcomes, at least when considered individually.

**Figure 1:**
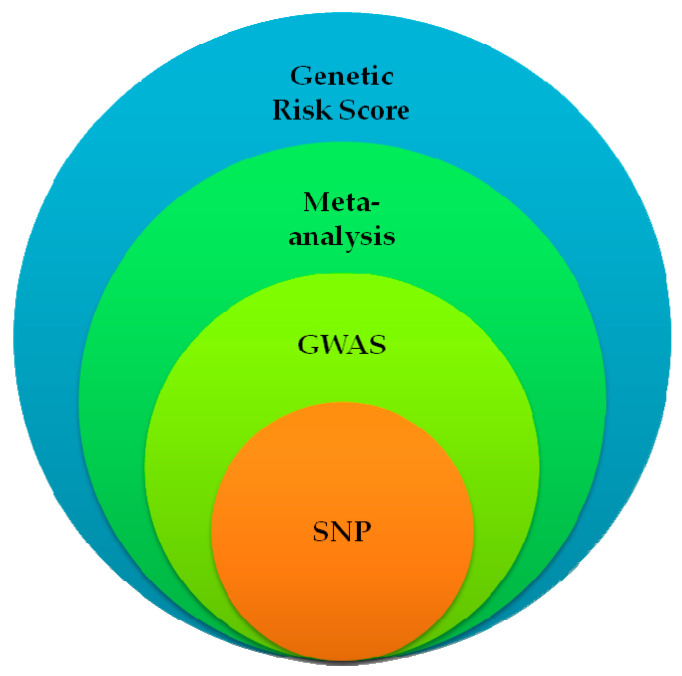
Scientific approach in revealing associations in disease genetic background.

A wider view on this application leads to combining candidate risk SNPs with other conservative and well-known clinical risk factors, creating a new study approach—the genetic risk score (GRS) (Figure 1).

Multiple individual genetic markers with small effect sizes may be used in combination to generate a greater effect size. The simultaneous use of the most common and strongest risk markers (like SNPs) and other clinical risk factors (like high LDL cholesterol, low HDL cholesterol, high blood pressure, family history, diabetes, smoking, etc.) may have the desired discriminatory accuracy to distinguish between diseased and healthy subjects.

A common genetic background in some diseases (i.e., autoimmune) provides us with an interesting study on generating a multilocal GRS based on common variants that have already been associated with CAD. An analysis that quantified heritability using updated genome-wide approaches estimated the heritability of CAD to be between 40 and 50% [27].

Many CAD-associated SNPs have been identified, but many of their functions are largely unknown. Although most loci identified seem to act through induction of atherosclerosis, little is known about the underlying mechanisms. The main part of the CAD-associated SNPs is in non-coding regions of the human genome. Studies indicate that the loci primarily exert their effect through regulation of nearby genes, but a large proportion of these genes have not previously been linked to CAD or risk factors for CAD [28,29].

Most CAD GWAS studies have been conducted in populations of European-Caucasian origin. Fewer but important studies have been conducted in East and South Asian and African populations. Most GWAS loci associated with CAD discovered in Europeans have only been confirmed in East Asian populations, with strong and significant correlations with other cardiovascular diseases. In general, these two populations share common risk factor loci [30,31].

With the development of GWAS, more than 230 SNPs associated with CAD have been identified [23,28,32,33,34,35,36]. The CAD risk loci identified include genes affecting lipoprotein metabolism, hypertension, and other CAD-related risk factors [2].

Elevated LDL levels are causally associated with increased CAD risk, according to studies of familial hypercholesterolemia involving mutations in the *LDLR* gene. Genetic variants in the *PCSK9*, *NPC1L1*, and *HMGCR* genes are associated with serum LDL levels and are considered predictors of CAD risk [37,38]. For many years, plasma triglyceride concentration was considered an independent risk factor for CAD. The primary metabolism of plasma triglycerides depends on lipoprotein lipase (LPL). Genetic variants associated with reduced LPL function are known to be associated with increased cardiovascular risk [39]. LPL activity is increased by apolipoprotein A5 (ApoA5) and inhibited by ApoC3 and angiopoietin-like proteins 3 and 4. Variants within the *APOC3* gene are associated with lower plasma triglyceride levels and CAD risk, while functional mutations in the *APOA5* gene have the opposite effects [40,41].

Genetic conditions, as well as inflammation, contribute to the development of CAD. Inflammation is a major factor in the development of CAD. Inflammatory cells, such as Th1 cells, CD4 + cells, macrophages, and dendritic cells, secrete cytokines actively involved in the disease process, which affect the arterial wall to promote lipoprotein retention and atherosclerotic plaque formation [42]. Several biomarkers of inflammation have been shown to be useful in predicting cardiovascular disease risk. Christiansen et al. suggested that a common variant at the *TOMM40/APOE* rs2075650 locus was significantly associated with lower levels of C-reactive protein (CRP) in patients with stable CAD; however, there was no association with other inflammatory genes (*APOA1, IL6R, MRAS* and *PLG*) [43].

In this article, we focus only on a small number of SNPs that are associated with the inflammation process and its outcome. There is good evidence that several of these genetic risk variants predispose to CAD through inflammatory pathways [28,44].

## 4. Transforming Growth Factor-β1

TGFβ1 is a protein encoded by the TGFβ1 gene located on chromosome 19 (19q13.1). TGFβ1 has received special attention due to its multiple roles in important pathological processes, such as stimulating chemotaxis of macrophages and fibroblasts, stimulating extracellular matrix synthesis, and causing abnormalities in vascular cell proliferation [45,46]. TGFβ1 can be secreted by several cell types, such as peripheral blood mononuclear cells, endothelial cells, macrophages, platelets, vascular smooth muscle cells (VSMCs), myofibroblasts, and renal cells [47]. TGFβ1 has a direct regulatory function on endothelial cells in the vessel wall, vascular smooth muscle cells, and the extracellular matrix. 

**Figure 2:**
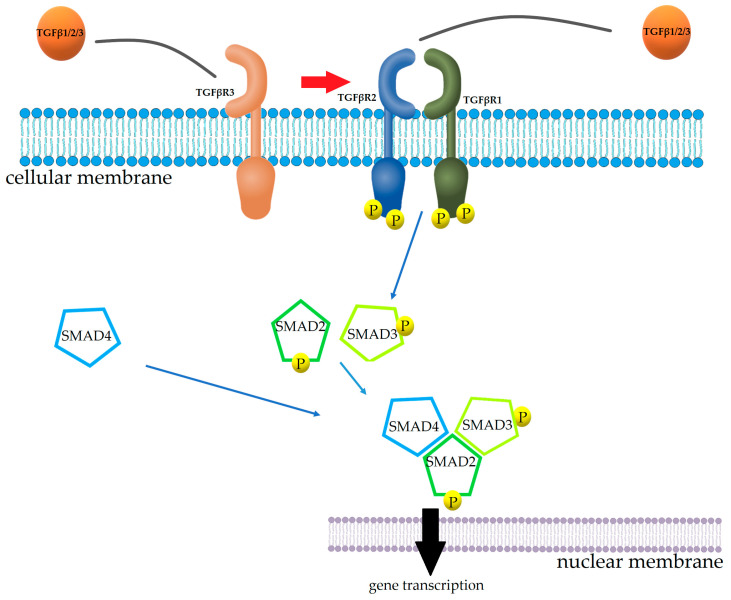
Schematic diagram of the SMAD TGFβ signalling pathway in vascular cells.

Intensive research into the mechanisms of TGFβ1 signalling over the past two decades has led to the development of a well-known signalling cascade. To initiate the TGFβ signalling pathway, TGFβ ligands must first bind to TGFβR2 on the cell surface. This binding results in the activation of TGFβR1, which leads to the phosphorylation of mothers against decapentaplegic homolog 2 (SMAD2) and SMAD3 proteins, which enable binding to SMAD4 and translocation to the cell nucleus. Nucleus SMAD proteins interact with other transcription factors to regulate transcription of target genes to control the cell response [46,47,48,49] (Figure 2). However, the exact mechanisms controlling the TGFβ1 signalling within the arterial vasculature are still incompletely understood.

TGFβ proteins play a key role in the development of myocardial fibrosis [50]. After myocardial infarction (MI), TGFβ signalling is altered and plays a key role in infarct healing and cardiac remodelling [51]. TGFβ1 and TGFβ2 are induced early after MI, while TGFβ3 shows delayed and prolonged regulation [52]. TGFβ has been described to act as a tumour suppressor in early carcinogenesis by inducing apoptosis or inhibiting cell growth, while promoting tumorigenesis and metastasis by suppressing local immune surveillance and promoting tumour progression in advanced stages of cancer [53,54].

TGFβ1 protein levels in the blood may not reflect normal TGFβ1 levels in the vascular interstitium, which are directly involved in the pathogenesis of CAD. Arterial wall fibroblasts send paracrine signals to VSMCs, causing macrophages to migrate toward the damaged area of the stented arterial wall in the presence of locally released TGFβ [55]. Platelets, which are involved in thrombus formation in the arterial wall, release large amounts of TGFβ1. Lower levels of serum TGFβ1 protein are generally considered useful biomarkers for the diagnosis and risk stratification of CAD [56,57]. Serum cytokine levels most often vary among populations, and their role in determining CAD is limited [58,59]. The fact that TGFβ1 levels in peripheral blood are genetically determined and depend on other inheritance mechanisms causes confusion about its independent role in CAD. Also, inconsistent results regarding the role of TGFβ1 levels in the development of CAD have been reported in studies of CAD in animal models [52]. Gichkun et al. hypothesized that the *TGFβ1* gene and its multiple variants are involved in creating a genetic predisposition to myocardial fibrosis in heart transplant recipients [60].

It has been proven that some polymorphisms within the *TGFβ1* gene can affect its expression and protein synthesis. Dysregulation of the TGFβ1 signalling pathway caused by a mutation can be associated with an increased risk of cardiovascular diseases. Tseng et al. analysed 54 *TGFβ* SNPs with a potential functional role in CAD (*TGFβ2*: 47 SNPs; *TGFβ3*: 7 SNPs); none of them remained statistically significant with an adjusted *p* < 0.05 [61]. 

The SNPs rs1800468 (800G/A) and rs1800469 (509C/T) are located in the gene’s promoter, while three other common SNPs—rs1800470 (29T/C), rs1800471 (913G/C) and rs1800472 (11929C/T)—are located in the coding regions [24]. These genetic variants are generally in strong linkage disequilibrium (LD) with each other [55]. Some studies suggest that these polymorphisms are associated with coronary atherosclerosis [24,25]. 

The mechanism for the effect of the rs1800469 polymorphism on the development of end-stage heart failure remains unclear. A study by Barsova et al. indicated a positive association between *TGFβ1* rs1800469 and a genetic predisposition to early MI in patients aged ≤ 50 years [58]. It is possible that the rs1800469 polymorphism alters promoter affinity for the transcription factors and inhibits TGFβ expression, thus activating proinflammatory cytokines (tumour necrosis factor α and interleukin 1) that contribute to the progression of CAD [58]. No association between the 509C/T polymorphism and CAD was detected in a group of German patients [60]. The inconclusive data from several studies on the rs1800469 promoter SNP still do not support its role in CAD.

One of the most studied genetic variants of *TGFβ1* is the rs1800470 polymorphism—a proline to leucine swap at codon 10 (Pro10Leu) of the protein [60,61,62,63]. The A allele of this SNP is known to be associated with elevated levels of TGFβ1 in peripheral blood [60]. The rs1800470 genotype has previously been associated with the risk of developing cardiovascular disease, including cerebral infarction [64], silent myocardial ischemia in patients with DM [65], and complications of CAD [66]. Interesting conclusions have come from observations of the Han population in China. Yang et al. suggested that gender may influence the role of TGFβR in CAD and the *TGFβ1* rs1800470 polymorphism was associated with CAD severity [67]. The frequency of the T allele was significantly and positively correlated with the number of stenosed coronary arteries (three or more vessels) [67]. In one of our previous studies, we examined the association between polymorphisms in the *TGFβ1* gene (rs1800469, rs1800470) and the risk of unstable angina and selected clinical parameters affecting CAD risk [66]; however, we did not find any significant risk factors for unstable angina in the Polish population. In a Mexican population, the rs1800470 polymorphism was associated with the risk of developing restenosis after coronary stent implantation [68]. Gichkun et al. analysed the genetic predisposition to myocardial fibrosis in heart transplant recipients and found a significant association with the rs1800470 polymorphism [60].

Studies have shown that the minor G allele of the *TGFβ1* rs1800471 polymorphism is associated with higher levels of gene expression and increased serum TGFβ1 protein levels [60,69,70]. Cambien et al. found that rs1800471 was associated with complications of CAD [71]. Morris et al., using a large meta-analysis, confirmed significant positive associations between the *TGFβ1* SNPs rs1800469, rs1800470, and rs1800471 and CAD complications [24]. These associations between SNPs and CAD were evaluated using additive, dominant, and recessive family inheritance models. Significant positive associations were observed between CAD complications and the minor alleles of rs1800469 (*p* = 0.031), rs1800470 (*p* = 0.021), and rs1800471 (*p* = 0.021). The odds ratios from this meta-analysis differed from the results of previous studies [24].

Other polymorphisms of the *TGFβ1* gene that affect its expression include the rs1982073 polymorphism. The results of a meta-analysis show an association between rs1800469 and rs1982073 in the *TGFβ1* gene and the risk of CAD in Caucasian populations, thus confirming that TGFβ1 signalling may be involved in the pathogenesis of CAD. Carriers of rare alleles of two genetic variants (rs1800469 and rs1982073) in *TGFβ1* have a 15% increased risk of CAD [26]. Koch et al. studied the rs1800469, rs1982073, rs1800471, and rs1800472 polymorphisms of the *TGFβ1* gene in patients with MI. A specific sequence of haplotypes was associated with MI in men, independent of age, hypertension, hypercholesterolemia, smoking, and diabetes. Interestingly, none of the genotypes or haplotypes were associated with MI in women, confirming that gender is an independent risk factor for CAD [72]. Table 1 presents the SNPs in *TGFβ* and *TGFβR* genes that are commonly analysed.

## 5. Transforming Growth Factor-β1 Receptor

TGFβ receptor (TGFβR) is a serine/threonine protein kinase present mainly on the cell surface. The receptor includes three isoforms: TGFβR1, TGFβR2, and TGFβR3 (the last one lacks kinase activity). TGFβ1-related signalling pathways are activated by the binding of TGFβ1 to its receptor [45,46].

The *TGFβR2* gene is located on chromosome 3 (3p22). It contains seven exons and encodes the TGFß type II receptor, a 565-amino-acid transmembrane tyrosine kinase with a molecular weight of about 60 kDa [76]. Genetic variations in TGFβR2 will produce different effects depending on the investigated tissue. *TGFβR2* is thought to be a tumour suppressor gene and to play a role in striated cell differentiation and coronary artery remodelling. Mutations in *TGFβR2* are associated with many cardiovascular diseases, such as Loeys–Dietz syndrome, familial thoracic aortic aneurysms, and sudden cardiac arrest in CAD patients [61,77,78]. In the cardiovascular system, it may lead to abnormal splicing of TGFβR2, resulting in loss of function of TGFß signalling activity in extracellular matrix formation and causing Marfan syndrome [77]. *TGFβR2* in the gastrointestinal tract acts as a tumour suppressor gene; approximately 30% of colorectal cancers carry various *TGFβR2* mutations [79]. 

TGFβ signalling is crucial for smooth muscle cell differentiation and contractile protein function. Mutations in TGFβR2 disrupt TGFβ signalling, creating genetic conditions that predispose to thoracic aortic aneurysm and dissection. Signalling through the TGFβR2 receptor in endothelial cells plays an important role in cardiac development and promotes fibrosis and myocardial remodelling in CAD [80]. 

Tseng et al. analysed 258 candidate SNPs with a potential functional role in CAD (*TGFβR1*: 24 SNPs; *TGFβR2*: 125 SNPs; *TGFβR3*: 109 SNPs), but only a single SNP in *TGFβR2* (rs9838682) remained statistically significant [61]. The rs9838682 polymorphism lies in a region of high LD in a block of haplotypes spanning more than 43,600 base pairs (bp). In our previous study, we examined the association between polymorphisms in the *TGFβR2* receptor gene (rs6785358, rs9838682) and CAD. We did not find any significant risk factors for unstable angina, but we were able to link the *TGFβR2* rs9838682 polymorphism with effects on lipid parameters in patients with CAD [66]. We observed increased values of total plasma cholesterol and LDL cholesterol, as well as triglycerides in patients with the *TGFβR2* rs9838682 AA genotype. The *TGFβR2* rs9838682 polymorphism was also associated with sudden cardiac arrest (SCA) [61]. The minor allele of rs6785358 in the Han population was associated with congenital heart defects [48], but not with CAD [64]. 

The study by Choi et al. compared the sequences of eleven SNPs in the *TGFβR2* gene between a group with Kawasaki disease and a control group. Three of them—rs1495592, rs6550004, and rs795430—were associated with the development of Kawasaki disease (KD). One SNP—rs1495592—was associated with coronary artery lesions (CALs) in the group with KD [74]. In another study, *TGFβR2* rs6550004 was associated with the development of KD, and rs1495592 was associated with coronary artery lesions in children with this disease [74,75]. Neither *TGFβR2* rs6785385 nor SNP rs764522 was associated with CAD severity in a Chinese population [73].

The study presented here has some limitations. We only analysed SNPs among *TGFβ1* and *TGFβR2* genes, not mentioning the numerous signalling pathways of other genes and proteins. In addition, we selected only the most common and intensively analysed SNPs without discussion of rare polymorphisms and insertion/deletion variants.

## 6. Conclusions

CAD is caused by reduced permeability of the coronary arteries due to atherosclerotic and thrombotic lesions, as well as ongoing inflammation in the coronary arteries. Previous studies have shown that numerous environmental and genetic factors are involved in the development of this disease. The main environmental factors preventing CAD include proper diet, adequate physical activity, and normal values of lipid and carbohydrate metabolism parameters. The recent years have brought many studies confirming the role of numerous genetic factors in the development of CAD. These include genes affecting lipid and carbohydrate metabolism, regulating endothelial and vascular smooth muscle function, influencing the coagulation system, or affecting the immune system. The inflammatory process occurring in the coronary vessels is very important in the development of CAD. It participates in the initiation of the atherosclerotic process by affecting the formation of foam cells and atherosclerotic plaques, and also affects the stability of the atherosclerotic plaque. The inflammation taking place in the atherosclerotic plaque is the cause of coronary occlusion and the occurrence of acute coronary syndromes. One important mediator of inflammation is TGFβ1. TGFβ1 plays an important role in the processes leading to CAD, such as stimulating chemotaxis of macrophages and fibroblasts, as well as increasing extracellular matrix synthesis. Previous studies have shown that polymorphisms within the *TGFβ1* gene are associated with the risk of developing various forms of CAD. Genetic risk factors, including SNPs, have been extensively studied in recent years, and considerable progress has been made to better understand their role in CAD, as well as the function of each new locus.

When evaluating CAD risk factors, it is important to keep in mind the multifactorial basis of this disease, including both environmental and genetic factors. *TGFβ1* polymorphisms are among the many genetic polymorphisms associated with the risk of developing CAD. The effect of single polymorphisms on CAD risk is small and should be considered along with other polymorphisms and other disease risk factors. Research on the role of genetic polymorphisms in the development of CAD not only contributes to the search for risk factors for the disease, but also contributes to a better understanding of its pathogenesis. Knowledge of the role of specific pro-inflammatory mediators in the development of CAD may enable the development of new therapeutic strategies for the treatment of CAD in the future, particularly the development of new antiplatelet drugs or the development of strategies to prevent stent occlusion. The complexity of the pathogenesis of CAD prompts us to continue further research to understand the numerous risk factors for this disease, the mechanisms affecting plaque development and regulating endothelial function, as well as the search for new therapeutic strategies.

Search Strategy and Inclusion Criteria: two independent authors (D.M. and A.P.) searched PubMed/MEDLINE/Embase from database inception until 1 February 2023, for GWAS and original studies regarding TGFβ and its receptor polymorphisms in heart impairments. The following search string was used in PubMed (TGFB* OR TGFβ* OR TGFBR* OR TGFβR* OR TGF-β* OR TGF-β* OR “transforming growth factor β”* OR “TGFß receptor”) AND (SNP OR “single nucleotide polymorphism” OR polymorphism OR gene) AND (“coronary artery disease” OR CAD OR “coronary heart disease” OR CHD OR “ischemic heart disease” OR IHD OR “myocardial infarction” OR MI). The exclusion criteria were as follows: (i) lack of association with TGFβ, and (ii) SNP citation in more than one publication.

## Figures and Tables

**Table 1:** Commonly analysed SNP variants in *TGFβ* and *TGFβR* genes.

	ID Number	Alias Name	Allele/Altered Allele	Gene Region	Reference(s)
*TGFB1*	rs1800468	−800G/A	G/A	Promotor	[24,26,71,73]
	rs1800469	−509C/T	C/T	Promotor	[24,26,63,64,65,66,71,72,73]
	rs1800471	913G/C	G/C	Coding region	[24,26,72,73]
	rs1800472	11929 C/T	C/T	Coding region	[24,26,72,73]
	rs1800470	+29T/C	T/C	Coding region	[24,59,60,63,64,65,66,67]
	rs1800820	−988C/A	C/A	5′ region	[71,73]
	rs1982073	+10T/C, 868 T/C	T/C	Signal peptide region	[26,72,73]
*TGFBR2*	rs6785358	−3779A/G	A/G	5′ upstream promoter region	[48,66,67]
	rs9838682	none	A/G	Introgenic	[61,66]
	rs1495592	none	C/T	Intron variant	[74,75]
	rs6550004	none	A/C	Intron variant	[74]
	rs795430	none	C/T	Introgenic	[74]
	rs764522	−1444C/G	C/G	5′ upstream promoter region	[48,67]

## Data Availability

Not applicable.

## References

[1] Nabel E.G., Bonow R.O., Mann D.L., Zipes D.P., Libby P. (2012). Principles of cardiovascular molecular biology and genetics. Braunwald’s Heart Disease. A Textbook of Cardiovascular Disease.

[2] McPherson R., Tybjaerg-Hansen A. (2016). Genetics of Coronary Artery Disease. Circ. Res..

[3] Hanson M.A., Fareed M.T., Argenio S.L., Agunwamba A.O., Hanson T.R. (2013). Coronary artery disease. Prim. Care.

[4] Gertler M.M., Garn S.M., White P.D. (1951). Young candidates for coronary heart disease. J. Am. Med. Assoc..

[5] Murabito J.M., Pencina M.J., Nam B.H., D’Agostino R.B., Wang T.J., Lloyd-Jones D., Wilson P.W.F., O’Donnell C.J. (2005). Sibling cardiovascular disease as a risk factor for cardiovascular disease in middle-aged adults. JAMA.

[6] Marenberg M.E., Risch N., Berkman L.F., Floderus B., de Faire U. (1994). Genetic susceptibility to death from coronary heart disease in a study of twins. N. Engl. J. Med..

[7] Muller C. (1938). Xanthomata, hypercholesterolemia, angina pectoris. Acta Med. Scand..

[8] Zdravkovic S., Wienke A., Pedersen N.L., Marenberg M.E., Yashin A.I., De Faire U. (2002). Heritability of death from coronary heart disease: A 36-year follow-up of 20 966 Swedish twins. J. Intern. Med..

[9] Palomaki G.E., Melillo S., Bradley L.A. (2010). Association between 9p21 genomic markers and heart disease: A meta-analysis. JAMA.

[10] Jarinova O., Stewart A.F., Roberts R., Wells G., Lau P., Naing T., Buerki C., McLean B.W., Cook R.C., Parker J.S. (2009). Functional analysis of the chromosome 9p21.3 coronary artery disease risk locus. Arterioscler. Thromb. Vasc. Biol..

[11] Zhou X., Han X., Wittfeldt A., Sun J., Liu C., Wang X., Gan L.-M., Cao H., Liang Z. (2016). Long noncoding RNA ANRIL regulates inflammatory responses as a novel component of NF-κB pathway. RNA Biol..

[12] Patel R.S., Schmidt A.F., Tragante V., McCubrey R.O., Holmes M.V., Howe L.J., Direk K., Åkerblom A., Leander K., Virani S.S. (2019). Association of Chromosome 9p21 with Subsequent Coronary Heart Disease Events. Circ. Genom. Precis. Med..

[13] Futuyma D.J. (2017). Evolution.

[14] McPherson R. (2009). A gene-centric approach to elucidating cardiovascular risk. Circ. Cardiovasc. Genet..

[15] Lewis C.M., Knight J. (2012). Introduction to genetic association studies. Cold Spring Harb. Protoc..

[16] Samani N.J., Erdmann J., Hall A.S., Hengstenberg C., Mangino M., Mayer B., Dixon R.J., Meitinger T., Braund P., Wichmann H.-E. (2007). Genomewide association analysis of coronary artery disease. N. Engl. J. Med..

[17] Helgadottir A., Thorleifsson G., Manolescu A., Gretarsdottir S., Blondal T., Jonasdottir A., Jonasdottir A., Sigurdsson A., Baker A., Palsson A. (2007). A common variant on chromosome 9p21 affects the risk of myocardial infarction. Science.

[18] McPherson R., Pertsemlidis A., Kavaslar N., Stewart A., Roberts R., Cox D.R., Hinds D.A., Pennacchio L.A., Tybjaerg-Hansen A., Folsom A.R. (2007). A common allele on chromosome 9 associated with coronary heart disease. Science.

[19] Ye S., Willeit J., Kronenberg F., Xu Q., Kiechl S. (2008). Association of genetic variation on chromosome 9p21 with susceptibility and progression of atherosclerosis: A population-based, prospective study. J. Am. Coll. Cardiol..

[20] Helgadottir A., Thorleifsson G., Magnusson K.P., Grétarsdottir S., Steinthorsdottir V., Manolescu A., Jones G.T., E Rinkel G.J., Blankensteijn J.D., Ronkainen A. (2008). The same sequence variant on 9p21 associates with myocardial infarction, abdominal aortic aneurysm and intracranial aneurysm. Nat. Genet..

[21] Smith J.G., Melander O., Lovkvist H., Hedblad B., Engstrom G., Nilsson P., Carlson J., Berglund G., Norrving B., Lindgren A. (2009). Common genetic variants on chromosome 9p21 confers risk of ischemic stroke: A large-scale genetic association study. Circ. Cardiovasc. Genet..

[22] Pearson T.A., Manolio T.A. (2008). How to interpret a genome-wide association study. JAMA.

[23] Nikpay M., Goel A., Won H.H., Hall L.M., Willenborg C., Kanoni S., Saleheen D., Kyriakou T., Nelson C.P., Hopewell J.C. (2015). A comprehensive 1000 Genomes-based genome-wide association meta-analysis of coronary artery disease. Nat. Genet..

[24] Morris D.R., Moxon J.V., Biros E., Krishna S.M., Golledge J. (2012). Meta-analysis of the association between transforming growth factor-beta polymorphisms and complications of coronary heart disease. PLoS ONE.

[25] Du L., Gong T., Yao M., Dai H., Ren H.G., Wang H. (2019). Contribution of the polymorphism rs1800469 of transforming growth factor β in the development of myocardial infarction: Meta-analysis of 5460 cases and 8413 controls (MOOSE-compliant article). Medicine.

[26] Lu Y., Boer J.M., Barsova R.M., Favorova O., Goel A., Müller M., Feskens E.J., on behalf of PROCARDIS CARDIoGRAM Consortium (2012). TGFB1 genetic polymorphisms and coronary heart disease risk: A meta-analysis. BMC Med. Genet..

[27] Won H.H., Natarajan P., Dobbyn A., Jordan D.M., Roussos P., Lage K., Raychaudhuri S., Stahl E., Do R. (2015). Disproportionate Contributions of Select Genomic Compartments and Cell Types to Genetic Risk for Coronary Artery Disease. PLoS Genet..

[28] Deloukas P., Kanoni S., Willenborg C., Farrall M., Assimes T.L., Ingelsson E., Saleheen D., Erdmann J., Goldstein B.A. (2013). Large-scale association analysis identifies new risk loci for coronary artery disease. Nat. Genet..

[29] Musunuru K., Strong A., Frank-Kamenetsky M., Lee N.E., Ahfeldt T., Sachs K.V., Li X., Li H., Kuperwasser N., Ruda V.M. (2010). From noncoding variant to phenotype via SORT1 at the 1p13 cholesterol locus. Nature.

[30] Khera A.V., Emdin C.A., Drake I., Natarajan P., Bick A.G., Cook N.R., Chasman D.I., Baber U., Mehran R., Rader D.J. (2016). Genetic Risk, Adherence to a Healthy Lifestyle, and Coronary Disease. N. Engl. J. Med..

[31] Marigorta U.M., Navarro A. (2013). High trans-ethnic replicability of GWAS results implies common causal variants. PLoS Genet..

[32] Coronary Artery Disease (C4D) Genetics Consortium (2011). A genome-wide association study in Europeans and South Asians identifies five new loci for coronary artery disease. Nat. Genet..

[33] Howson J.M.M., Zhao W., Barnes D.R., Ho W.K., Young R., Paul D.S., Waite L.L., Freitag D.F., Fauman E.B., Salfati E.L. (2017). Fifteen new risk loci for coronary artery disease highlight arterial-wall-specific mechanisms. Nat. Genet..

[34] Nelson C.P., Goel A., Butterworth A.S., Kanoni S., Webb T.R., Marouli E., Zeng L., Ntalla I., Lai F.Y., Hopewell J.C. (2017). Association analyses based on false discovery rate implicate new loci for coronary artery disease. Nat. Genet..

[35] Schunkert H., König I.R., Kathiresan S., Reilly M.P., Assimes T.L., Holm H., Preuss M., Stewart A.F., Barbalic M., Gieger C. (2011). Large-scale association analysis identifies 13 new susceptibility loci for coronary artery disease. Nat. Genet..

[36] Musunuru K., Kathiresan S. (2019). Genetics of Common, Complex Coronary Artery Disease. Cell.

[37] Cohen J.C., Boerwinkle E., Mosley T.H., Hobbs H.H. (2006). Sequence variations in PCSK9, low LDL, and protection against coronary heart disease. N. Engl. J. Med..

[38] Ference B.A., Majeed F., Penumetcha R., Flack J.M., Brook R.D. (2015). Effect of naturally random allocation to lower low-density lipoprotein cholesterol on the risk of coronary heart disease mediated by polymorphisms in NPC1L1, HMGCR, or both: A 2 × 2 factorial Mendelian randomization study. J. Am. Coll. Cardiol..

[39] Nordestgaard B.G., Varbo A. (2014). Triglycerides and cardiovascular disease. Lancet.

[40] Jørgensen A.B., Frikke-Schmidt R., Nordestgaard B.G., Tybjærg-Hansen A. (2014). Loss-of-function mutations in APOC3 and risk of ischemic vascular disease. N. Engl. J. Med..

[41] Do R., Project N.E.S., Stitziel N.O., Won H.H., Jørgensen A.B., Duga S., Merlini P.A., Kiezun A., Farrall M., Goel A. (2015). Exome sequencing identifies rare LDLR and APOA5 alleles conferring risk for myocardial infarction. Nature.

[42] Libby P., Ridker P.M., Hansson G.K. (2011). Progress and challenges in translating the biology of atherosclerosis. Nature.

[43] Christiansen M.K., Larsen S.B., Nyegaard M., Neergaard-Petersen S., Ajjan R., Würtz M., Grove E.L., Hvas A.-M., Jensen H.K., Kristensen S.D. (2017). Coronary artery disease-associated genetic variants and biomarkers of inflammation. PLoS ONE.

[44] Huan T., Zhang B., Wang Z., Joehanes R., Zhu J., Johnson A.D., Ying S., Munson P.J., Raghavachari N., Wang R. (2013). A systems biology framework identifies molecular underpinnings of coronary heart disease. Arterioscler. Thromb. Vasc. Biol..

[45] Schaan B.D., Quadros A.S., Sarmento-Leite R., De Lucca G., Bender A., Bertoluci M. (2007). ‘Correction’: Serum transforming growth factor beta-1 (TGF-beta-1) levels in diabetic patients are not associated with pre-existent coronary artery disease. Cardiovasc. Diabetol..

[46] Grainger D.J. (2007). TGF-beta and atherosclerosis in man. Cardiovasc. Res..

[47] Pardali E., Goumans M.J., ten Dijke P. (2010). Signaling by members of the TGF-beta family in vascular morphogenesis and disease. Trends Cell Biol..

[48] Huang F., Li L., Shen C., Wang H., Chen J., Chen W., Chen X. (2014). Association between TGFBR2 gene polymorphisms and congenital heart defects in Han Chinese population. Nutr. Hosp..

[49] Heldin C.H., Miyazono K., ten Dijke P. (1997). TGF-beta signalling from cell membrane to nucleus through SMAD proteins. Nature.

[50] Lijnen P.J., Petrov V.V., Fagard R.H. (2000). Induction of cardiac fibrosis by transforming growth factor-beta(1). Mol. Genet. Metab..

[51] Bujak M., Frangogiannis N.G. (2007). The role of TGF-beta signaling in myocardial infarction and cardiac remodeling. Cardiovasc. Res..

[52] Dewald O., Ren G., Duerr G.D., Zoerlein M., Klemm C., Gersch C., Tincey S., Michael L.H., Entman M.L., Frangogiannis N.G. (2004). Of mice and dogs: Species-specific differences in the inflammatory response following myocardial infarction. Am. J. Pathol..

[53] Seoane J., Gomis R.R. (2017). TGF-β Family Signaling in Tumor Suppression and Cancer Progression. Cold Spring Harb. Perspect. Biol..

[54] Xia L., Xiao X., Liu W.L., Song Y., Liu T.J.J., Li Y.J., Zacksenhaus E., Hao X.J., Ben-David Y. (2018). Coactosin-like protein CLP/Cotl1 suppresses breast cancer growth through activation of IL-24/PERP and inhibition of non-canonical TGFβ signaling. Oncogene.

[55] Suthanthiran M., Li B., Song J.O., Ding R., Sharma V.K., Schwartz J.E., August P. (2000). Transforming growth factor-beta 1 hyperexpression in African-American hypertensives: A novel mediator of hypertension and/or target organ damage. Proc. Natl. Acad. Sci. USA.

[56] Wang S., Zhang Q., Wang Y., You B., Meng Q., Zhang S., Li X., Ge Z. (2018). Transforming Growth Factor β1 (TGF-β1) Appears to Promote Coronary Artery Disease by Upregulating Sphingosine Kinase 1 (SPHK1) and Further Upregulating Its Downstream TIMP-1. Med. Sci. Monit..

[57] Ser Ö.S., Çetinkal G., Kiliçarslan O., Dalgıç Y., Batit S., Keskin K., Özkara G., Aslan E.I., Aydoğan H.Y., Yıldız A. (2021). The comparison of serum TGF-beta levels and associated polymorphisms in patients with coronary artery ectasia and normal coronary artery. Egypt. Heart J..

[58] Barsova R.M., Titov B.V., Matveeva N.A., Favorov A.V., Sukhinina T.S., Shahnovich R.M., Ia R.M., Favorova O.O. (2012). Contribution of the TGFB1 Gene to Myocardial Infarction Susceptibility. Acta Nat..

[59] Brusentsov D.A., Nikulina S.Y., Shesternya P.A., Chernova A.A. (2018). Association of RS1800470 polymorphic variants of the transforming growth factor β1 (TGF-β1) gene with the severity of coronary atherosclerosis. Rus. J. Cardiol..

[60] Gichkun O.E., Shevchenko O.P., Kurabekova R.M., Mozheiko N.P., Shevchenko A.O. (2021). The rs1800470 Polymorphism of the TGFB1 Gene Is Associated with Myocardial Fibrosis in Heart Transplant Recipients. Acta Nat..

[61] Tseng Z.H., Vittinghoff E., Musone S.L., Lin F., Whiteman D., Pawlikowska L., Kwok P.Y., Olgin J.E., Aouizerat B.E. (2009). Association of TGFBR2 polymorphism with risk of sudden cardiac arrest in patients with coronary artery disease. Heart Rhythm.

[62] Stadtlober N.P., Flauzino T., Santos L.F.D.R.F., Iriyoda T.M.V., Costa N.T., Lozovoy M.A.B., Reiche E.M.V., Simão A.N.C. (2021). TGFB1 +869 T > C (rs1800470) variant is independently associated with susceptibility, laboratory activity, and TGF-β1 in patients with systemic lupus erythematosus. Autoimmunity.

[63] Paradowska-Gorycka A., Roszak M., Stypinska B., Lutkowska A., Walczyk M., Olesinska M., Wajda A., Piotrowski P., Puszczewicz M., Majewski D. (2019). IL-6 and TGF-β gene polymorphisms, their serum levels, as well as HLA profile, in patients with systemic lupus erythematosus. Clin. Exp. Rheumatol..

[64] Tao H.M., Chen G.Z., Cheng G.P., Shan X.Y. (2012). The haplotype of the TGFβ1 gene associated with cerebral infarction in Chinese. Can. J. Neurol. Sci..

[65] Cruz M., Fragoso J.M., Alvarez-León E., Escobedo-de-la-Peña J., Valladares A., Juárez-Cedillo T., Pérez-Méndez O., Vargas-Alarcón G. (2013). The TGF-B1 and IL-10 gene polymorphisms are associated with risk of developing silent myocardial ischemia in the diabetic patients. Immunol. Lett..

[66] Malinowski D., Safranow K., Pawlik A. (2023). TGF-β1 and TGFβR2 Gene Polymorphisms in Patients with Unstable Angina. Biomedicines.

[67] Yang M., Zhu M., Tang L., Zhu H., Lu Y., Xu B., Jiang J., Chen X. (2016). Polymorphisms of TGFβ-1 and TGFBR2 in relation to coronary artery disease in a Chinese population. Clin. Biochem..

[68] Fragoso J.M., Zuñiga-Ramos J., Arellano-González M., Alvarez-León E., Villegas-Torres B.E., Cruz-Lagunas A., Delgadillo-Rodriguez H., Peña-Duque M.A., Martínez-Ríos M.A., Vargas-Alarcón G. (2015). The T29C (rs1800470) polymorphism of the transforming growth factor-β1 (TGF-β1) gene is associated with restenosis after coronary stenting in Mexican patients. Exp. Mol. Pathol..

[69] Rao M., Guo D., Jaber B.L., Tighiouart H., Pereira B.J., Balakrishnan V.S. (2004). Transforming growth factor-beta 1 gene polymorphisms and cardiovascular disease in hemodialysis patients. Kidney Int..

[70] Nikolova P.N., Ivanova M.I., Mihailova S.M., Myhailova A.P., Baltadjieva D.N., Simeonov P.L., Paskalev E.K., Naumova E.J. (2008). Cytokine gene polymorphism in kidney transplantation--impact of TGF-beta 1, TNF-alpha and IL-6 on graft outcome. Transpl. Immunol..

[71] Cambien F., Ricard S., Troesch A., Mallet C., Générénaz L., Evans A., Arveiler D., Luc G., Ruidavets J.-B., Poirier O. (1996). Polymorphisms of the transforming growth factor-beta 1 gene in relation to myocardial infarction and blood pressure. The Etude Cas-Témoin de l’Infarctus du Myocarde (ECTIM) Study. Hypertension.

[72] Koch W., Hoppmann P., Mueller J.C., Schömig A., Kastrati A. (2006). Association of transforming growth factor-beta1 gene polymorphisms with myocardial infarction in patients with angiographically proven coronary heart disease. Arterioscler. Thromb. Vasc. Biol..

[73] Peng Z., Zhan L., Chen S., Xu E. (2011). Association of transforming growth factor-β1 gene C-509T and T869C polymorphisms with atherosclerotic cerebral infarction in the Chinese: A case-control study. Lipids Health Dis..

[74] Choi Y.M., Shim K.S., Yoon K.L., Han M.Y., Cha S.H., Kim S.K., Jung J.H. (2012). Transforming growth factor beta receptor II polymorphisms are associated with Kawasaki disease. Korean J. Pediatr..

[75] Shi C.P., Zhang H.Y. (2013). Association of single nucleotide polymorphism in TGFBR2 gene with Kawasaki disease and coronary artery lesions. Chin. J. Contemp. Pediatr..

[76] Lin H.Y., Wang X.F., Ng-Eaton E., Weinberg R.A., Lodish H.F. (1992). Expression cloning of the TGF-beta type II receptor, a functional transmembrane serine/threonine kinase. Cell.

[77] Mizuguchi T., Collod-Beroud G., Akiyama T., Abifadel M., Harada N., Morisaki T., Allard D., Varret M., Claustres M., Morisaki H. (2004). Heterozygous TGFBR2 mutations in Marfan syndrome. Nat. Genet..

[78] Baas A.F., Medic J., van’t Slot R., de Kovel C.G., Zhernakova A., Geelkerken R.H., E Kranendonk S., van Sterkenburg S.M., E Grobbee D., Boll A.P. (2010). Association of the TGF-beta receptor genes with abdominal aortic aneurysm. Eur. J. Hum. Genet..

[79] Muñoz N.M., Upton M., Rojas A., Washington M.K., Lin L., Chytil A., Sozmen E.G., Madison B.B., Pozzi A., Moon R.T. (2006). Transforming growth factor beta receptor type II inactivation induces the malignant transformation of intestinal neoplasms initiated by Apc mutation. Cancer Res..

[80] Robinson P.N., Arteaga-Solis E., Baldock C., Collod-Béroud G., Booms P., De Paepe A., Dietz H.C., Guo G., A Handford P., Judge D.P. (2006). The molecular genetics of Marfan syndrome and related disorders. J. Med. Genet..

